# Analyzing the impact of clay minerals on the reservoir quality of the Lower Goru Formation using Unsupervised Machine Learning

**DOI:** 10.1371/journal.pone.0324793

**Published:** 2025-05-22

**Authors:** Kausar Noreen, Tahir Azeem, Faisal Rehman, Yawar Amin, Qazi Adnan Ahmad, Mureed Hussain

**Affiliations:** 1 Department of Earth Sciences, Quaid-i- Azam University, Islamabad, Pakistan; 2 College of Energy and Mining Engineering, Shandong University of Science and Technology, Qingdao, China; 3 Department of Soil and Environmental Sciences, Ghazi University, Dera Ghazi Khan, Pakistan; Maria Curie-Sklodowska University: Uniwersytet Marii Curie-Sklodowskiej, POLAND

## Abstract

The reservoir quality of the Lower Goru Formation is highly variable due to its heterogeneous nature influenced by sea level fluctuations during the Early Cretaceous period. This study applies an unsupervised machine learning workflow, integrating Principal Component Analysis (PCA) for dimensionality reduction, Self-Organizing Maps (SOM) for clustering, and fuzzy classification for geological labeling, alongside petrophysical evaluation and cross-plot analysis, to assess the impact of clay minerals on the reservoir quality of the Lower Goru Formation in the NIM-Tay block, Lower Indus Basin, Pakistan. Petrophysical analysis delineates a potential reservoir zone (1455–1517 m) characterized by 13.9% effective porosity and 27.3% water saturation. The first four principal components explain approximately 90% of the dataset variance. Electrofacies classification distinguishes four facies—Impermeable Reservoir, Potential Reservoir, Non-Reservoir, and Tight Reservoir—each corresponding to specific clay mineral assemblages. Cross-plot and electrofacies analysis reveal that facies dominated by chlorite and montmorillonite preserve porosity (15%) and permeability (888.87 mD), whereas kaolinite-rich and mixed-layer clay facies significantly reduce reservoir quality. This study provides a reproducible and scalable framework for integrating machine learning with petrophysical workflows, offering improved reservoir characterization not only in the Lower Indus Basin but also in similar heterogeneous sandstone reservoirs globally.

## Introduction

Reservoir characterization, which comprises the prediction of a variety of features that may be used to characterize and assess the reservoir, is an imperative phase in the exploration and development of hydrocarbon reserves [1]. A complete integration of quantitative sedimentological and mineralogical data with petrophysical interpretation is necessary for a successful reservoir description [2]. The ability to identify different kinds of clay minerals and lithofacies is also a crucial aspect of reservoir characterization [3]. Porosity and permeability are two primary characteristics that have a significant impact on reservoir quality [4] and are influenced by the lithological composition and occurrence of various kinds of clay minerals [3,5–7].

In the presence of clay minerals, the development of clastic reservoirs for hydrocarbon resources is one of the most important and challenging problems [6,7]. In reservoir rocks, clay minerals exist in a variety of shapes and compositions, either as primary or diagenetic products [7]. These minerals have a significant impact on various petrophysical parameters, including effective porosity, permeability, and hydrocarbon saturation [8]. Clay minerals are fine-grained materials that are categorized into four broad classes based on their crystal structure: kaolinite, chlorite, illite, and smectite (montmorillonite) [9,10]. Each clay group can affect reservoir rock qualities differently.

Hence, understanding the distribution and composition of clay minerals is critical for reliable reservoir quality evaluations. Smectite (montmorillonite) and kaolinite commonly reduce porosity and permeability by clogging pore throats or swelling when exposed to water [11,12]. Kaolinite often occurs as pore-filling or vermicular assemblages [13,14], while montmorillonite can either reduce reservoir quality through swelling or, in certain cases, preserve permeability when coating pores [6,7]. Chlorite coatings are especially important, as they inhibit quartz overgrowth and preserve primary porosity and permeability [6,7,15–18]. In contrast, illitic clays typically form pore-bridging fabrics that obstruct fluid flow and decrease permeability [19].

Well-log analysis is one of the most widely used techniques in the oil and gas industry, and geoscientists often utilize it to describe reservoir properties [20,21]. By statistically characterizing subsurface formations, well log data offer ground truth from which various connections may be constructed [22]. Nevertheless, the conventional well-log analysis is difficult, time-consuming, and reliant on the expertise of the person analyzing the data [3,20,21,23–25]. In recent years, machine learning has developed as an innovative method in the field of geosciences to estimate expected output based on pattern identification in available data [26,27]. Incomplete well log data restricts reservoir characterization and hinders automation, which can be attributed to logging difficulties, tool restrictions, or missing variables [28–30]. Workflows are often subjective and inefficient because expert geoscientists frequently use time-consuming procedures to create training data, improve forecasts, and validate low-confidence outputs [20,21,31]. Although they struggle with data heterogeneity and quality inconsistencies, algorithmic approaches for good correlation do solve certain problems [28,32,33].

Due to their inefficiency and poor precision, traditional statistical approaches frequently fail [20,21,34]. Manual methods for estimating missing data and fixing measurements take a lot of effort and are prone to mistakes [20,35,36]. These difficulties limit the choice of models and impede the use of data [34,37]. A workable answer is provided by machine learning, which uses well log measurements to categorize subsurface geology and predict lithofacies. Automated clustering and classification methods improve efficiency, scalability, and reliability by avoiding the drawbacks of manual methods [31,34]. These techniques improve reservoir analysis and characterization by using indirect connections between geological characteristics and well logs. The machine learning approaches increase reservoir characterization precision by detecting subtle mineralogical variations [38], whereas sophisticated algorithms improve porosity and permeability predictions driven by clay mineral composition [39]. Data-driven models give a more complete picture of reservoir heterogeneity and quality, allowing for a more precise evaluation. Additionally, incorporating machine learning minimizes human bias and improves consistency in geological evaluations [40]. Machine learning uses predictive analytics to optimize reservoir development plans by precisely assessing mineralogical impacts, thereby enhancing subsurface exploration decision-making. These points make the machine learning approach favorable over traditional well logging methods in terms of reliability and resolution.

Machine learning employs a variety of techniques for data analysis, including clustering, classification, and regression [24]. Unsupervised and supervised techniques are the two main subcategories of machine learning [24,25,41,42]. The supervised learning approach requires prior characterization of the facies categories, which is commonly performed by identifying unique lithologies in cores [41]. Unsupervised machine learning, on the other hand, primarily relies on the anticipated values and input parameters [24,43,44]. It automatically identifies the hidden patterns between inputs and establishes groups of similar data points without any guidance. The identified patterns help to classify the formations into producing and non-producing intervals, ultimately improving the reservoir characterization and modeling [45].

**Fig 1:**
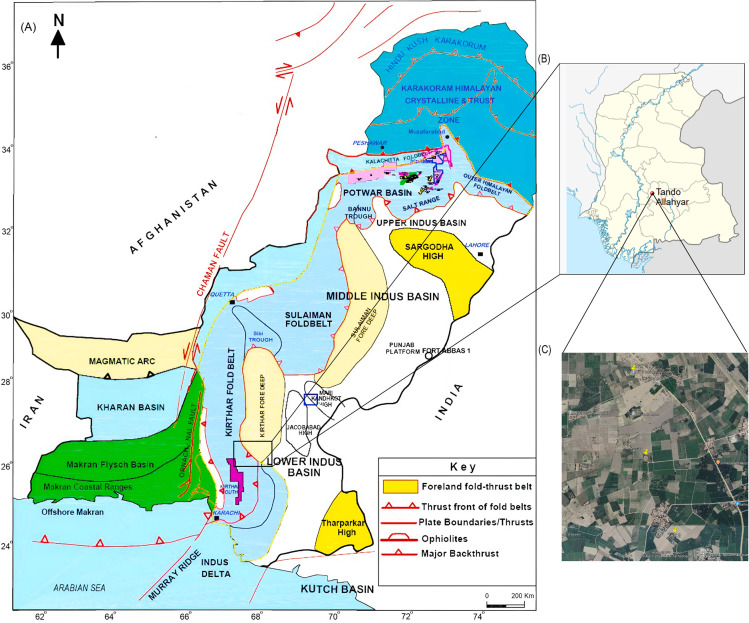
Map showing the regional geology and tectonics of the study area. **(A)** Tectonic Map of Pakistan. **(B)** Location of study area. **(C)** Satellite image showing the location of study wells (modified after [59]).

The NIM-TAY block is tectonically situated in the Lower Indus Basin, Pakistan (Fig 1). In this region, the Lower Goru Formation of the Early Cretaceous age is a recognized reservoir that produces hydrocarbons [46]. Although the Lower Goru Formation is a known reservoir rock, its quality is affected by its heterogeneity. The primary cause of the heterogeneity found in the Lower Goru Formation is the presence of different diagenetic and depositional settings [47–49]. Diagenetic alterations frequently affect porosity and permeability. These properties are mainly controlled by the features formed during deposition, including detrital grains size, their sorting, and the mineralogical composition of the reservoir rocks [50].

A number of authors have studied the Lower Goru Formation and addressed the existence of various types of clay minerals and their effect on the reservoir rock through conventional methods [5,49,51,52]. [5] analyzed the Lower Goru Formation from the Kadanwari area of the Lower Indus Basin based on cross-plot techniques to determine lithologies and clay minerals. They found that the Lower Goru Formation in the area consisted of sandstone interbedded with carbonates and shales with the key clay minerals such as chlorite, kaolinite, and montmorillonite. Petrophysical analysis confirmed good reservoir zones along with the presence of clay minerals affecting the reservoir quality. [49] investigated the impact of diagenesis and associated clay minerals on the reservoir quality of the Lower Goru Formation by utilizing core studies in the Lower Indus Basin. Authigenic chlorite has been found to be responsible for preserving the primary porosity while recrystallization of chlorite has destroyed the pore spaces [49]. [51] investigated the role of “porosity-preserving” chlorite cements in maintaining the reservoir quality at greater depths in the Sawan area of Lower Indus Basin. Chlorite cementation and chlorite rims were found to be the main reason behind the high porosity and permeability even at greater depths. [52] also studied the diagenetic controls on the reservoir quality of the Lower Goru Formation mainly focusing on porosity and permeability variations within the basal sands through core and well log studies. Two types of porosities have been discussed: primary porosity due to chlorite coatings, and secondary porosity due to feldspar and volcanic rock dissolution. The quartz and calcite cementation along with illite and kaolinite as diagenetic clay minerals are responsible for porosity and permeability reduction.

Despite these valuable insights, conventional methods face limitations when dealing with the complex heterogeneity of the Lower Goru Formation. Due to the extremely heterogeneous behavior of the Lower Goru Formation, the reservoir quality abruptly changes within the vicinity of Lower Indus Basin, thus making it very difficult and time consuming to analyze the formation through traditional well logging methods. Machine learning approaches account for the heterogeneity and provide accurate results in complex geological reservoirs [53,54]. As previously mentioned, machine learning offers an effective solution because the model is trained based on common well logs and can be applied to larger areas, thereby reducing time and minimizing ambiguities associated with manual or traditional methods.

In this study, we analyzed the potential reservoir interval, evaluated the presence of different clay minerals, and investigated their impact on the quality of the Lower Goru Formation in the NIM-TAY block for the first time. The selected methodology integrates petrophysical and cross-plot analysis for potential zone demarcation and clay mineral identification, combined with unsupervised machine learning techniques for electrofacies classification based on well log data. The machine learning workflow includes Principal Component Analysis (PCA) for data dimensionality reduction, Self-Organizing Maps (SOM) for clustering, and fuzzy classification for facies labeling. Although several researchers have applied machine learning techniques in the vicinity of the Lower Indus Basin [25,43,53,55–57] and similar formations nearby [58], the combination of PCA, SOM, and fuzzy classification for electrofacies classification of clay minerals is applied for the first time in this study area. Based on the methodology outlined above, this study aims to answer the following research questions.

- Does the SOM and fuzzy classification suggest the heterogeneous nature of the Lower Goru Formation?
- What is the implication of petrophysical analysis in conjunction with the electrofacies classification for the number of facies in the Lower Goru Formation?
- Are there any clay minerals in the Lower Goru Formation and do they have an impact on the reservoir quality of the formation?

## Geology and tectonics

**Fig 2:**
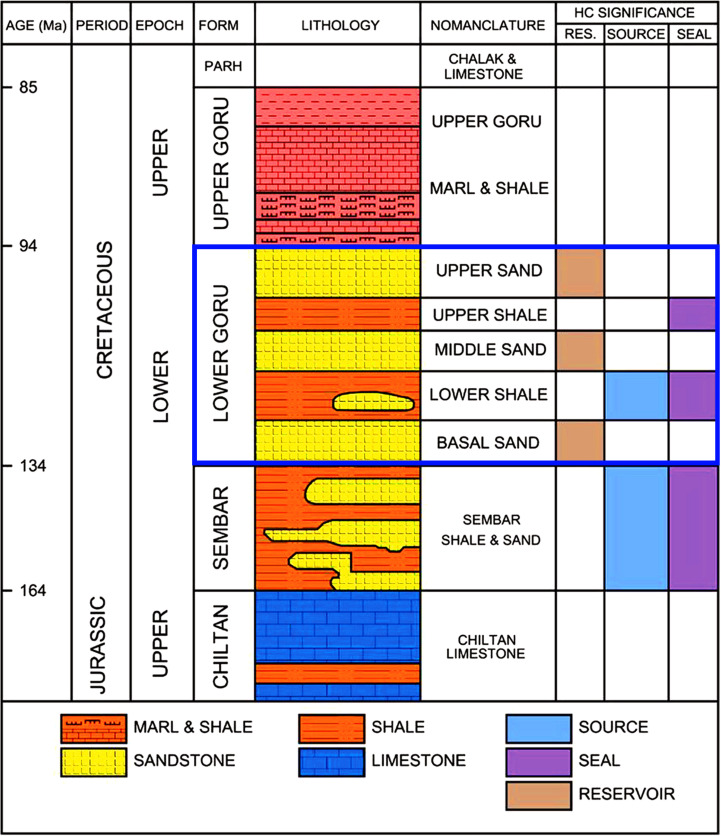
The generalized stratigraphic column of the Lower Indus Basin. The target formation has been highlighted with blue rectangle [63–65].

The Indus Basin of Pakistan is one of the biggest sedimentary basins in the world and a hotspot for hydrocarbon exploration. It is further divided into three parts: the Upper, Middle, and Lower Indus Basins based on the structural characteristics [60–62]. On the northern side of the Lower Indus Basin, the Suleiman Fold-Belt and Central Indus Basin are located while Kirthar Fold-Belt is situated on the western side (Fig 1). Clastic and carbonate deposits in this area range from the Infra-Cambrian era to the present (Fig 2). It lies in the tectonic zone known as the “Indus Platform and Foredeep,” which has a number of structural features [61].

During the lower Cretaceous, a major tectonic activity occurred in this region, creating a rift zone, which resulted in the development of the Lower Indus Basin [66]. It belongs to the extra continental trough down-warped basins which are the product of continuous extensional stresses during the creation of the region’s major tectonic formations [67,68]. The horst and grabens, which are frequently found in the study area, are signs of the extensional tectonic regime that predominated during the split of the Indo-Pak peninsula. The dipping direction of sedimentary layers is from east to west on a regional scale. These structures play a significant part in the buildup of hydrocarbons. In the Lower Indus Basin, many reservoirs of sandstone dating from the Cretaceous to the Paleogene have been discovered and their analysis suggests a substantial hydrocarbon potential [69].

The study area (NIM-TAY block) is located in the district Tando Allah Yar of Hyderabad Division, Sindh Province, Pakistan (Fig 1). Geologically, it is located south of the Southern Sindh Monocline and is a part of the Lower Indus Basin [70]. The most significant packages in this region are the Lower Goru and the Sembar formations [70]. The main source rock in the study area is the Sembar Formation of Early Cretaceous age [71] (Fig 2). Geochemical investigations of the Sembar Formation in the vicinity of study area shows that it contains up to 3.5% thermally mature organic material. The hydrocarbons in the Lower Goru Sands are considered to be mostly sourced from the Sembar Formation [72]. It mainly consists of shale, with siltstone and sandstone serving as subsidiary components. The Sembar Formation was deposited in marine environments throughout much of the Greater Indus Basin, and its thickness varies from 0 to over 260 m [73]. A fair amount of total organic carbon content is found in the Sembar Formation throughout the basin [74].

The Lower Goru Formation of Early Cretaceous is the most prolific reservoir in the study area [75]. The two different lithological parts of the Goru Formation are the Upper Goru Formation, which contains more shales with distinctive sandstone, and the Lower Goru Formation, which mainly comprises sand layers [60,76,77]. The Lower Goru Formation has five members in the study area, namely, Upper Sand, Upper Shale, Middle Sand, Lower Shale, and Basal Sand [72]. The primary reserves of hydrocarbons are the upper sands of the Lower Goru Formation, exhibiting excellent porosity and permeability values. The effective porosity values are in the range of 11–26% with high concentration of hydrocarbon saturation ranging from 65–81% [78]. Sandy layers act as reservoir rock; however, the reservoir characteristics quickly change within the area after a few kilometers [60]. Lithological variability of these rocks is mostly caused by variations in the sediment supply and environmental factors [77]. Primary seal in this area is the thick shale package of the Upper Goru Formation [71] (Fig 2).

## Data set and methodology

### Data set

**Fig 3:**
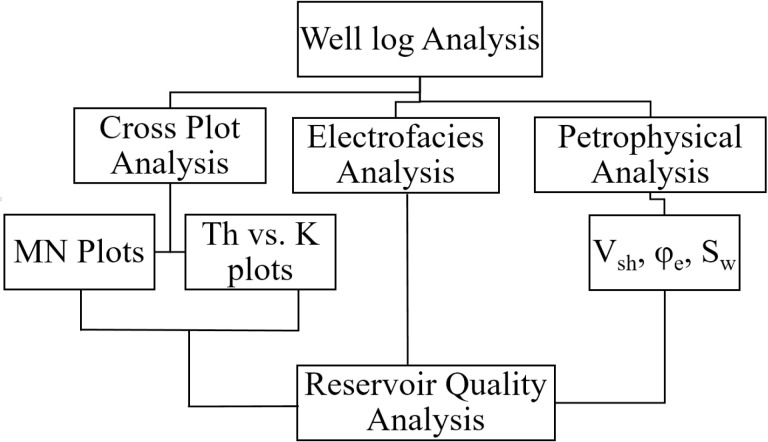
The workflow adopted in the current research to study the impact of minerals on the reservoir quality of the Lower Goru Formation.

This study was carried out in different steps by utilizing the wireline logs data from three wells namely, Tando Allah Yar 01, Tando Allah Yar 02, and Tando Allah Yar 03 (Fig 3). The data was acquired from Directorate General of Petroleum Concessions (DGPC), Pakistan. Information details of the wells are listed in Table 1. Available wireline log data includes neutron porosity (NPHI), density (RHOB), sonic (DT), gamma ray (GR), thorium (THOR), potassium (POTA), uranium (URAN), photo absorption Index (PEF), caliper, spontaneous potential and resistivity logs (shallow and deep, i.e., LLS, LLD).

**Table 1:** Shows the technical well data information for all three wells.

Technical Well Data			
Operator	OGDCL	Province	Sindh
Type	Development	Status	Gas
**Well Bore Name**	Tando Allah Yar-01		
**Longitude**	68.7433° E	Latitude	25.4244° N
**Elevation(m):**	20.6	Total Depth(m)	1750m
**Depth Reference**	KB		
**Well Bore Name**	Tando Allah Yar-02		
**Longitude**	68.7473° E	Latitude	25.4156° N
**Elevation(m):**	21.37	Total Depth(m)	1678m
**Depth Reference**	KB		
**Well Bore Name**	Tando Allah Yar-03		
**Longitude**	68.7418° E	Latitude	25.4349° N
**Elevation(m):**	20.15	Total Depth(m)	1610m
**Depth Reference**	KB		

Tando Allah Yar-01 well was selected to perform a detailed log analysis. It has a total depth of 1752m (Table 1). The Lower Goru Formation was encountered at 1447m and has a total thickness of 305m. The aforementioned logs have been acquired through the entire depth of the Formation. For petrophysical analysis, caliper, SP, GR, RHOB, NPHI, LLS, LLD logs were utilized whereas for M-N cross-plot analysis RHOB, DT and NPHI logs were used. The clay minerals were identified by means of THOR vs POTA ratio chart using the spectral Gamma ray logs, i.e., Thorium and Potassium. For unsupervised machine learning, the input logs used were GR, PEF, RHOB, POTA, THOR and URAN.

GR, RHOB, and PEF are fundamental petrophysical logs that provide critical insights into lithological variability. Specifically, GR measurements are indicative of shale content, RHOB reflects matrix and fluid densities, and PEF assists in mineralogical identification. The spectral gamma ray logs, namely, POTA, THOR, and URAN provide a refined discrimination of clay types and depositional environments, thereby enhancing the precision of mineralogical assessment [79,80]. These logs were therefore selected based on their ability to differentiate lithofacies and corresponding minerals within the Lower Goru Formation. Although alternative logs such as LLD and DT were available, they were deliberately excluded to reduce potential redundancy and avoid introducing strong correlations that could bias the unsupervised classification results.

The analysis was carried out in detail on well Tando Allah Yar-01 and the results are applied to the other two wells through an unsupervised machine learning approach. The step-by-step illustration of each technique is discussed in the next sections.

### Methods

#### Petrophysical analysis.

The study was initiated through petrophysical analysis along with MN cross-plots to determine the lithological and mineralogical composition. Different types of clay minerals were identified by Th vs. K cross-plots. Petrophysical analysis bridges the gap between seismic and core data and is a crucial step for reservoir characterization [46]. Typically, petrophysical parameters are determined in laboratories using core data and by examining data from drill logs [81]. Wireline logs offer an effective method for identifying hydrocarbon-saturated rocks in the absence of core samples [69]. The precise determination of further petrophysical characteristics, such as porosity and water saturation in shale formations, depends on the exact calculation of shale content [46].

**Fig 4:**
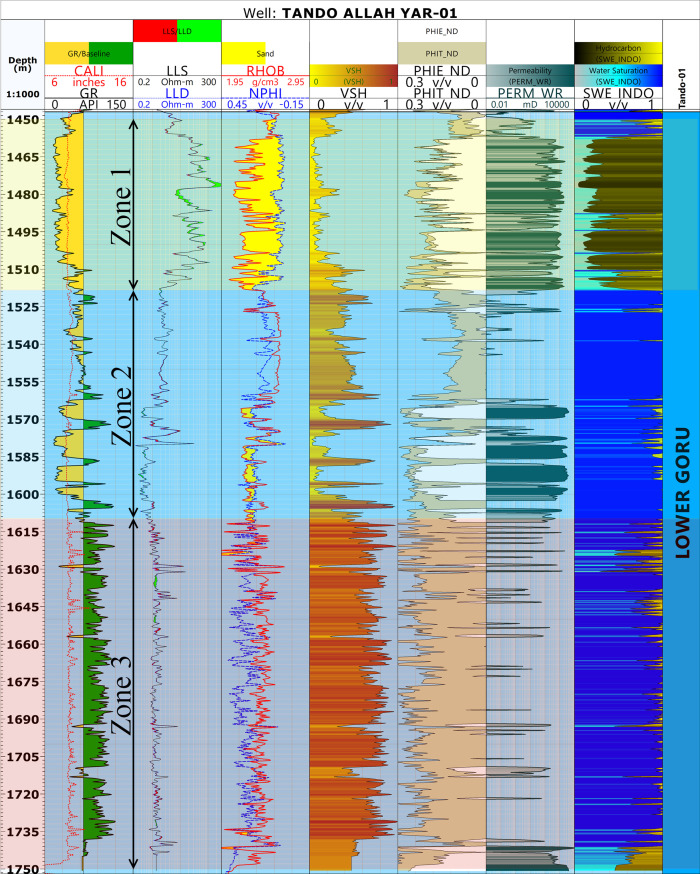
Petrophysical analysis of well Tando Allah Yar-01 indicating three marked zone based on different log responses. The first zone is a potential reservoir, the second is predominantly sand with shale interbedding leading to a diminished reservoir quality. The third bed is mainly comprised of shale beds and lacks any potential.

Different petrophysical parameters, i.e., shale volume, effective porosity, and water saturation were calculated following the workflow outlined in [46] and [82]. For permeability, unfortunately, the available data did not have the necessary core data. Therefore, the Wyllie-Rose equation given by [83] was used to calculate the permeability values as proposed by [82]. The results obtained are shown in Fig 4.

#### Cross-plots analysis (MN and Th vs. K).

**Fig 5:**
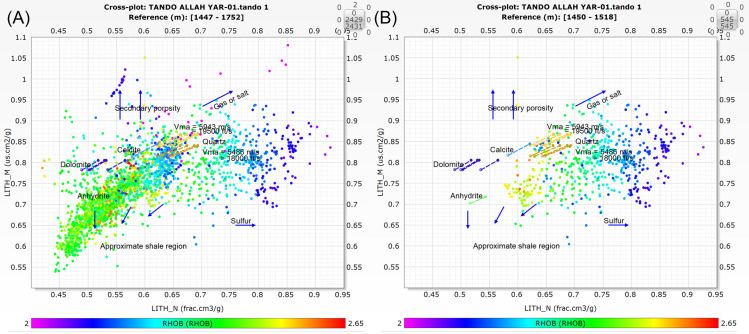
MN cross-plot for well Tando Allah Yar-01. (**A**) depicting the values for entire depth range of Lower Goru Formation. (**B**) depicting the values for Reservoir zone.

Cross-plot analyses of density–porosity logs are frequently used to identify the lithology of a formation, while natural gamma ray and neutron porosity logs are equally sensitive tools for lithological interpretation [84]. MN cross-plots are commonly used to identify a formation’s principal lithology (minerals), with pure rock minerals based on distinct points on the plot [85]. These points are termed as M and N on the plot, which are estimated using density, neutron, and sonic logs. As a result, when these two extents are plotted together, the lithology becomes more apparent [82]. We employed the methodology utilized by [5,82], and [3] by using different empirical equations for M and N values on the basis of different logs responses such as neutron porosity (NPHI), sonic (DT) and bulk density (RHOB) logs. These equations lead to the required results shown in Fig 5.

**Fig 6:**
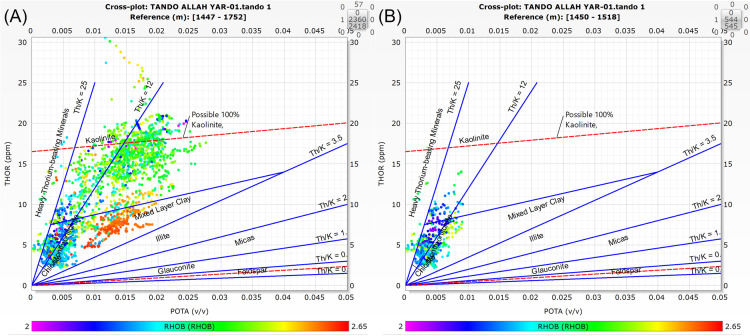
Th vs. K cross-plot for well Tando Allah Yar-01. (**A**) depicts values for the entire depth range of Lower Goru Formation (**B**) depicts the values for reservoir zone.

The spectral gamma ray logs are crucial for determining the presence and concentration of clay minerals in reservoir rocks [86]. Various cross-plots are in practice for the identification of different types of clay minerals such as Th vs. K, K vs. PEF, Th/K vs. PEF and Th/U vs. Th/K ratio. An accurate way to assess the different kinds of mineral found in clay is to use the cross-plot relationship between Th and K. Different clay minerals exhibit varying concentrations of Th and K and can therefore be segregated. To identify different types of clay minerals in the Lower Goru Formation, Th vs. K cross-plot was generated using the Thorium vs.Potassium cross-plot technique adopted from [3,5] (Fig 6).

#### Electrofacies analysis through unsupervised machine learning approach.

**Fig 7:**
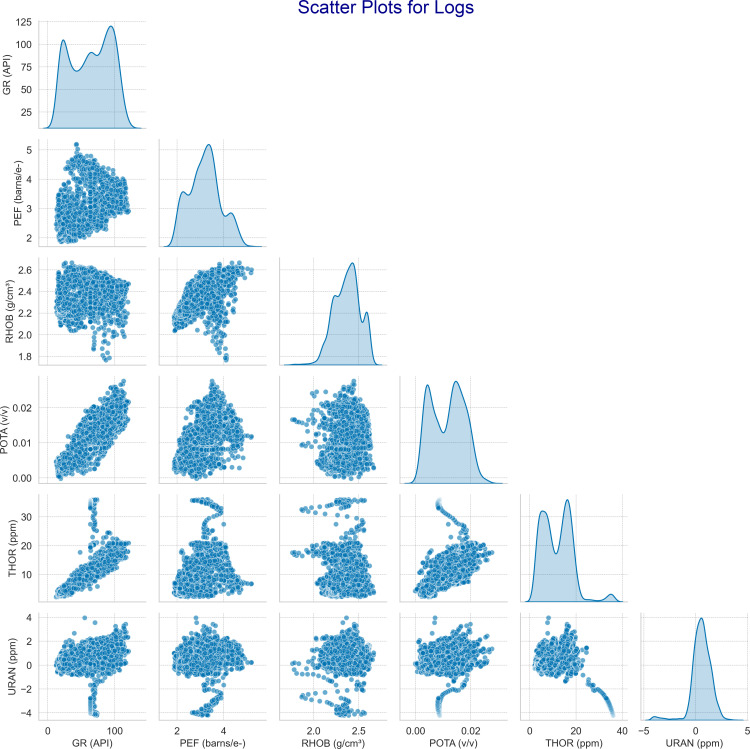
Pairwise scatter and distribution plots of input well logs used for electrofacies classification. The figure displays interrelationships between GR (API), PEF(b/e^-^), RHOB (g/cm^3^), POTA(v/v), THOR (ppm), and URAN (ppm). The scatter plots highlight patterns and correlations between variables, while the diagonal curves represent the smoothed distribution of each log. These inherent associations support the application of PCA for dimensionality reduction prior to clustering.

Electrofacies is a useful technique that utilizes the well log data to distinguish multiple facies within a formation [48]. In this study, the unsupervised machine learning method was selected to classify electrofacies based on clay minerals. Initially, well Tando Allah Yar 01 was selected for classification. Afterward, the other two wells were incorporated to study the lateral variations in clay minerals throughout the area. The data was pre-processed using PCA, and subsequently clustered and labeled through SOM and fuzzy classification. The initial step in PCA involves selecting the well logs data. The data is formatted as a matrix, represented as  Xn×p , with n  log curves for each of the p variables. The PCA algorithm reconstructs the data from the original features and as a result, removes the inherent correlations between the features [58]. The primary goal of PCA is to decrease the number of dimensions in the data set without significantly reducing the amount of information. The resulting principal components (PCs) are linear combinations of the original variables, ranked by the amount of variance they capture [87]. The interrelationship among the original features is shown in the scatter plot (Fig 7).

The equation given by [88] is used to calculate the variance of each well log curve. Variance is the average squared deviation of all its n values, calculated using the formula in Eq. 1:


$$Vi=\frac{1}{n-1}\sum {\mathrm{\backslash nolimits}}_{m=1}^{n}{\left(x_{im\;\;\;\;\;\;\;}-\;\;\;\;\;\;\;{\bar{x}}_{i}\right)}^{2}$$ (1)

In this case, m represents the object, n is the total number of log curves, ${\bar{x}}_{i}$ is the mean value of i, and, $x_{im\;\;\;\;\;\;\;}$ is the value of the ith variable in the object m. According to [89], covariance measures the influence of one variable on another and can be calculated using Eq. 2:


$$Cij=\;\;\;\;\;\;\;\frac{1}{n-1}\sum {\mathrm{\backslash nolimits}}_{m=1}^{n}\left(x_{im}-\;\;\;\;\;\;\;{\bar{x}}_{i}\right)(x_{jm}-\;\;\;\;\;\;\;{\bar{x}}_{j})\;\;\;\;\;\;\;$$ (2)

Here, Cij is the covariance between variables i  and j. $x_{im}$ and $x_{jm}$ are the values of i and j (variables) in the m object while, ${\bar{x}}_{i}$ and ${\bar{x}}_{j}\;$ are the mean value of i and j.

Covariance and variance are useful for examining the differences in the dataset. These two are correlated, and the principal components are determined by calculating the cross-product of covariance. The data matrix is then subjected to Eigen analysis. Eigen vectors represents the direction of maximum variance, while Eigen values represent the magnitude of variance in those particular directions [90]. The following equation yields these values:


|A−   λI |   _0 (3)

where, A is the data set matrix, I is the identity matrix, and    λ is an Eigen value. PCA analysis resulted in the generation of various graphs and maps depicting the variability coverage of individual PCs, along with their cumulative coverage trend and contribution of individual features (Figs 8 and 9A).

**Fig 8:**
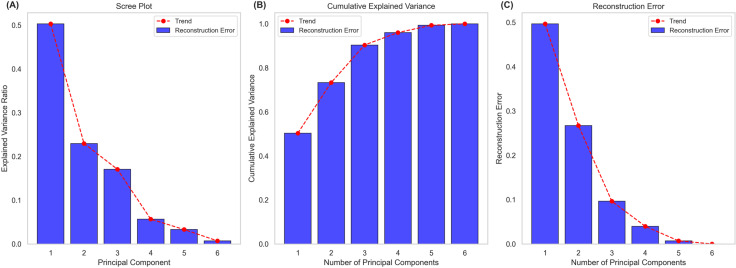
Evaluation of PCs selection using three diagnostic plots depicting the coverage of variability by individual PCs and their cumulative coverage trend. **(A)** Scree plot showing proportion of variance for each PC, with a sharp decline after PC4 indicating diminishing returns for further components. **(B)** Cumulative variance plot depicting that approximately 90% of the variance is captured by the first four components. **(C)** Reconstruction error plot for the fidelity of PCA-reconstructed data to the original features, illustrating minimal error reduction beyond PC4. These trends collectively justify the retention of the first four components for subsequent clustering analysis.

**Fig 9:**
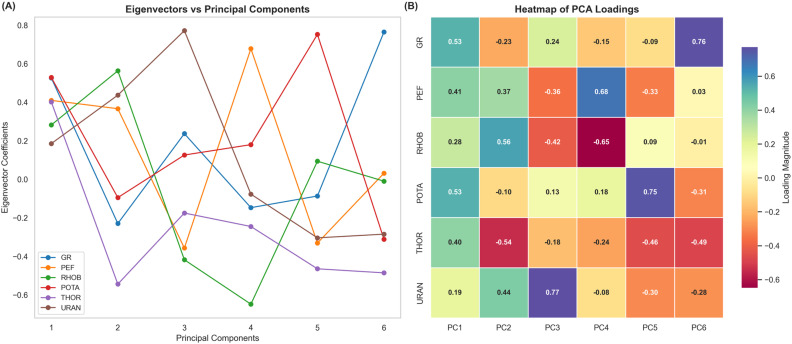
Contribution of input log variables in the PCs. **(A)** Line plot showing eigenvector coefficients (loadings) for each variable (GR, PEF, RHOB, POTA, THOR, URAN) across the six PCs, indicating the directional influence and relative contribution of each feature. **(B)** Heatmap of PCA loadings illustrating a visual summary of feature influence of each PC, with color intensity representing the magnitude of each feature within the respective PC. Together, these plots highlight the influence of features on each PC, offering interpretive clarity on variable relationships and dimensional structure in the dataset.

In the next step, a heatmap was generated for all PCA loadings (Fig 9B). The heatmap provides a color-coded visualization, making it easier to identify patterns where certain features are heavily weighted in specific principal components (PCs) [53,91,92]. The deeper hues and distinct contrasts, which emphasize each feature’s contribution, provide an intuitive understanding of the feature-PC relation [93].

After preprocessing the data using PCA, the next step was the application of a self-organizing map (SOM), also known as Kohonen maps [94]. SOM is mainly used in unsupervised machine learning applications mainly for dimensionality reduction and clustering [53]. In this step, the main PCs produced were cross plotted for cluster analysis using SOM. To ensure consistency in the input data during SOM training, each input feature was standardized using a linear transformation, and equal weighting (weight = 1) was assigned across all variables to avoid biasing the classification toward any single log. The dimensionality-reduced dataset from PCA was then used as input for clustering. The SOM was configured with unsupervised fuzzy classification, where initial cluster centers were determined using Ward’s hierarchical clustering method. A weighting factor of 1.2 was applied to regulate the fuzziness level of class boundaries, and the number of classes was set to five. These parameters were selected to strike a balance between resolution and interpretability of the electrofacies, based on prior geological understanding of the formation.

Clustering divides a dataset into distinct, outwardly separated, and internally homogenous groups by using a measure of similarity and dissimilarity between the groupings [87]. In SOM, nodes ranging from a few hundreds to thousands, connected to neighboring nodes are arranged on a regular low-dimensional grid [25]. The training phase and the mapping phase are the two primary parts of SOM. A square grid of neurons is used to initialize the network during the training phase. Each neuron is linked to a weight vector with the same dimensions as the input vector. To start the learning process, these weight vectors are first given random values [53]. Equation 4 from [53] was applied to summarize the process of SOM.


Wv(s+1)=Wv(s)+θ(u,v,s)*α(s)*[D(t)−Wv(s) (4)

**Fig 10:**
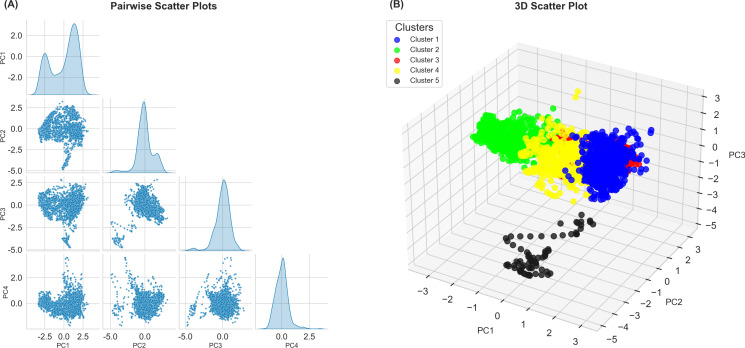
Principal Component scatter plots used for facies separation. **(A)** Pairwise scatter plots of the first four PCs (PC1–PC4), which together account for approximately 90% of the total variance. The spatial distribution and separation of data points across different PC combinations indicate latent patterns suitable for unsupervised clustering. **(B)** 3D scatter plot of the first three PCs, showing five discrete, color-coded clusters derived from geological interpretation. These visually distinguishable groups validate the effectiveness of PCA in enhancing separability for electrofacies classification.

In this equation, Wv stands for the current weight vector, s is current iteration, t is the targeted input data vector, D(t) is the vector, v is the node index on the map, u is the best matching unit (BMU) index on the map, α is the learning restraint brought on by the iteration progress, and θ(u,v,s) is the distance from the BMUs. [53]. At the end of the training process, the sample data is assigned to their BMU in the SOM grid [94]. The clustered data are shown in Fig 10 as a pairwise scatter plot (Fig 10A) and a 3D scatter plot (Fig 10B).

The fuzzy classification along with SOM defines the electrofacies through a self-interactive map that is clustered via SOM and labeled through fuzzy classification. In fuzzy classification, each node is randomly assigned to each group with a specific probability [95]. The procedure completes in three steps; in the first step, it calculates each group’s barycenter, weighted by the likelihood that each point belongs to the group, mathematically it can be expressed as:


$$\mu_{k}=\sum {\mathrm{\backslash nolimits}}_{i=1}^{n}P_{ik}^{\frac{1}{QQ-1}}\;\cdot \;x_{i}$$ (5)

Here, $P_{ik}$ is the probability that the point i   relates to the group k. The number of groups is defined by the class number parameter, $\mu_{k}$ is the barycenter of the group k and QQ   is used as the weighting factor. In the second step, new probabilities based on the separation between each node and its barycenter are computed using Eq. 6:


$$p_{nk}=\sum {\mathrm{\backslash nolimits}}_{i=1}^{nbclasses}{\left(\frac{ecart_{in}}{ecart_{kn}}\right)}^{\frac{1}{QQ-1}}$$ (6)

Here, $p_{nk}$ is the probability that the point n belongs to the group k and $ecart_{kn}=x_{n}-{\mu_{k}}^{2}$: which is the distance between the point n  and the barycenter of the group k. Convergence testing is completed in the third step. This method examines if the new and old probabilities differ from one another. If so, the updated probabilities are used. Otherwise, the model’s output is the new probabilities. The class of each point is determined by the highest likelihood. This process concludes in a series of results which are discussed in detail in section 4.3. The end product of SOM and fuzzy classification represents electrofacies in the form of a log, which can be easily differentiated on the log’s response.

## Results

### Petrophysical analysis

The petrophysical characteristics of the Lower Goru Formation were analyzed in detail based on the well log response of Tando Allah Yar-01 (Fig 4). The depth of the Lower Goru Formation ranges from 1447 to 1752 meters with a thickness of 305 meters. Furthermore, the Gamma Ray (GR) Log response indicates a gradual increase in shale volume from top to bottom. Based on the GR, RHOB, NPHI and resistivity logs response, the Lower Goru Formation is broadly divided into three zones with depth ranges of 1455-1517m, 1519-1610m, and 1611-1750m. (Fig 4). The first zone is the potential hydrocarbon producing zone as indicated by the separation between the resistivity logs (LLD and LLS), crossover of NPHI and RHOB, low shale volume, high effective porosity and permeability, and low water saturation (Fig 4). The second zone is majorly sand and shows some signs of potential reservoir, but also contains shale beds which resulted in diminished reservoir quality. This interpretation is primarily suggested by GR and shale volume log responses. Slight separation of resistivity logs and crossover of NPHI and RHOB in certain portions of the zone further supports this assessment. The third zone is predominantly composed of shale resulting in a low reservoir potential. This can be observed through a high GR and shale volume, high NPHI, and low effective porosity and permeability (Fig 4).

In Zone 1, the average values for shale volume, effective porosity, permeability, and water saturation were calculated as 15.4%, 13.9%, 434.7mD and 27.3%, respectively (Table 2). The petrophysical parameters indicate that this zone is favorable for hydrocarbon accumulation and can be marked as a reservoir zone with good effective porosity and permeability values. The large crossover between neutron porosity and density indicates the presence of gas (Fig 4). Zone 2 (1519–1610 m) indicates the presence of interbedded shale layers. These shale layers have disturbed the reservoir quality. The average values for different parameters in this zone were calculated as 35.8%, 10.2%, 719.53 mD, and 97.5% for shale volume, effective porosity, permeability, and water saturation, respectively (Table 2). In this zone, the effective porosity value decreased, while the shale volume and water saturation show an increase indicating significantly reduced reservoir quality due to the presence of shale. The occurrence of shale layers acts as a barrier, significantly reducing the hydrocarbon saturation. In the lower portion (1611–1750 m), the false crossover between the neutron porosity and density logs, high shale volume, water saturation and extremely low values of effective porosity and permeability indicate the presence of non-reservoir rock. The averages for this zone were calculated as 62.5%, 9.1%, 285.47 mD, and 92.2% for shale volume, effective porosity, permeability, and water saturation, respectively.

**Table 2:** Petrophysical analysis of Well Tando Allah Yar-01 suggesting three zones.

ZONES	Depth (m)	Average Volume of Shale (%)	Average Effective Porosity (%)	Average Permeability (mD)	Average Water Saturation (%) ()
Zone 1	1455-1517	15.4	13.9	434.7	27.3
Zone 2	1519-1610	35.8	10.2	719.53	97.5
Zone 3	1611-1750	62.5	9.1	285.47	92.2

### Cross-plot analysis

The lithological and mineralogical configuration of the Lower Goru Formation is inferred from MN cross-plot analysis (Fig 5). MN cross-plot combines density and neutron log information and offers detailed insights into lithology, porosity, and fluid content. The MN cross-plots are generated for both the entire depth of the Lower Goru Formation (Fig 5A) and the reservoir zone (Fig 5B) to examine the distribution and grouping of data points. The cross-plot analysis shows that the most data points lie within the quartz-rich zone and shale region, indicating that the core lithology of the formation is sandstone and shale with minor amounts of calcite, dolomite, and anhydrates (Fig 5A). Furthermore, the MN cross-plot at the reservoir level reveals that the major data clusters are in the quartz rich zone, particularly concentrated in the gas saturated zone characterized by low density values (Fig 5B). This confirms the findings of the petrophysical analysis that the reservoir zone is saturated with gas. Overall, the MN cross-plot results are consistent with those of the petrophysical analysis.

Since both petrophysical and MN cross-plot analyses show that the Lower Goru Formation consists of both sand and shale bodies, the presence of clay minerals within shale layers is also evident. Certain clay minerals significantly influence the petrophysical properties such as porosity and permeability negatively while others preserve them. This makes the identification of clay type a crucial step in reservoir characterization and quality prediction studies. The Th vs K cross-plot technique was utilized for the clay mineral identification across the entire formation depth of the Lower Goru Formation (Fig 6A) and within the reservoir zone interval (Fig 6B). The cross-plot analysis indicates the presence of chlorite, kaolinite, montmorillonite, and mixed layer clays (Fig 6A). Kaolinite and mixed layer clays exhibit high density values, while chlorite and montmorillonite are on the lower side (Fig 6A). However, in the reservoir zone, the concentration of chlorite with some traces of montmorillonite were observed (Fig 6B). The absence of micas and feldspars, along with the presence of the above-mentioned minerals indicate the abundance of “thorium-rich minerals” in the Lower Goru Formation.

### Electrofacies analysis

Electrofacies classification for clay minerals was performed based on unsupervised machine learning. This method allows for the gradual progression of facies transitions, which are often more realistic than traditional classification methods. The technique uses PCA to pre-process the data and reduce its dimensionality.

Initially, the logs were selected for data preprocessing. A scatter plot was created to study the correlation between input parameters. The plot reveals a clear pattern, indicating potential correlation between the input features. This is particularly evident for some logs namely, GR and POTA, while clustering tendencies are observable for the others (Fig 7). This preliminary analysis suggests the presence of inherent associations among the features, making the dataset viable for PCA and clustering.

PCA was applied based on the selected features and projected the data into 6 principal components (PCs). The scree plot illustrates the proportion of variance for each PC (Fig 8A). PC1 captures about 50% of the data variance, followed by PC2 with 25%. PC3 accounts for approximately 18–20% of the variance in the data. A noticeable flattening at PC4 indicates diminishing returns for further components (Fig 8A). This implies that the first four PCs are sufficient to represent the variability of majority of the dataset, thus justifying the use of these components dimensionality reduction.

Fig 8B shows the cumulative variance plot depicting the cumulative control of PCs to the total variance. Each bar emphasizes the individual contribution of PCs, while the trendline depicts the cumulative tendency of the captured explained variance. Over 90% variance is captured by PC4, thereby reinforcing the initial implication to retain the first four components (Fig 8B).

The reconstruction error plot is shown in Fig 8C, which quantifies the fidelity of reconstructed data from PCA relative to the original features. A steep line from PC1 to PC4 depicts that most of the variability is covered by these components (Fig 8C). Error reduction is minimal after PC4 suggesting that the later components contributes very slightly to the variance, thus further validating the selection of four components.

The resultant PCA components, i.e., eigenvectors, were rated based on how well they explained the value variances [96]. Fig 9A depicts the eigenvector coefficients, also known as loadings, for each feature across all six PCs. These values provide a detailed insight into the contribution of each feature to the PCs with higher absolute coefficient for a given PC giving a more significant contribution to the variance of that PC [97,98]. For PC1, GR and POTA have high positive loadings, indicating that the variance captured by PC1 is primarily derived from these features (Fig 9A). PC2 is mainly influenced by RHOB with the highest loading value. Similarly, URAN dominates PC3 with the highest eigenvector coefficient whereas PC4 is dominated by PEF with a strong positive loading value (Fig 9A). Notably, RHOB also has a high absolute loading value in PC4. POTA also has a strong association with PC5 indicating its unique contribution to this specific component. On the other hand, THOR shows notable influence with high absolute loading in PC1, PC5, and PC6 (Fig 9A). The distinct contribution of each feature to different PCs shown by the variations highlights the multivariate nature of the dataset.

Heat map shows consistency with the eigenvector plot, GR depicts the highest loading in PC1 with a value of 0.53, reinforcing its dominance in this component (Fig 9B). PC2 is dominated by RHOB with a value of 0.68. URAN dominates PC3 with a value of 0.77, whereas PEF has the highest magnitude of 0.68 in PC4. POTA contributes significantly to PC5 with a magnitude of 0.75 (Fig 9B).

**Fig 11:**
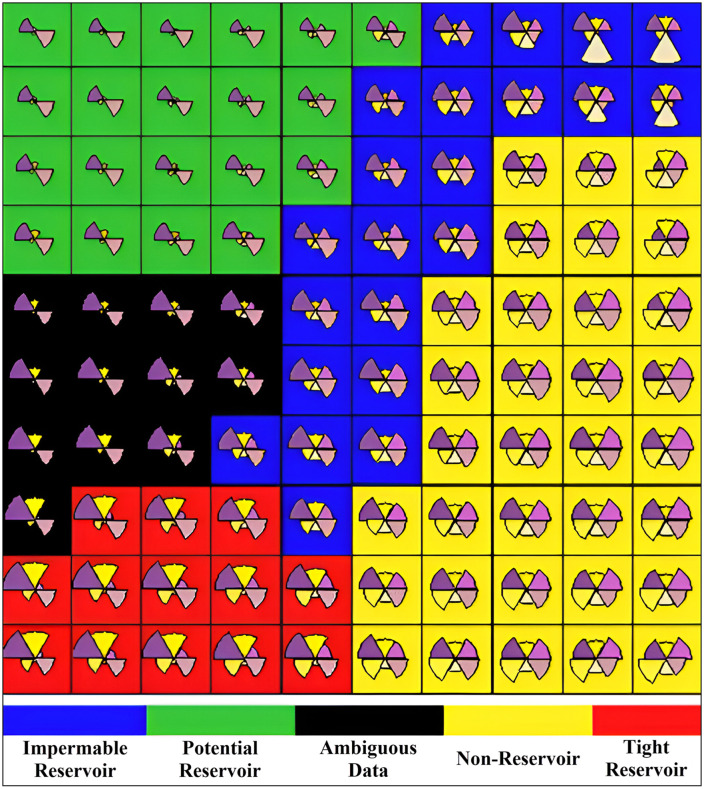
Electrofacies map generated using SOM combined with fuzzy classification, based on multi-log input data from all three wells. The five electrofacies are color-coded as: Impermeable Reservoir (blue), Potential Reservoir (green), Non-Reservoir (yellow), Tight Reservoir (red), and Ambiguous Data (black). Each neuron in the SOM grid represents a cluster of similar log responses, illustrated with embedded pie charts that summarize normalized multi-log signatures. The spatial arrangement of electrofacies highlights the model’s ability to capture subtle lithological variations, enabling robust facies classification for reservoir evaluation.

Clustering patterns between different pairs of PCs can be observed from the pairwise scatter plot (Fig 10A). Distinct groupings, particularly between PC1 and PC2, suggest inherent subgroups in the form of clusters. The density plots along the diagonal provide insights into the distribution of each principal component, with PC1 showing a bimodal distribution (Fig 10A). The data points from the first three PCs can be observed in the 3D scatter plot (Fig 10B). Based on the geological information and the required number of facies, the data was segregated into five clusters with each cluster illustrated with a distinct color. After pre-processing and selection of the first four PCs, SOM was applied to the data for the wells Tando Allah Yar-01, 02 and 03. The data was labeled using fuzzy classification (Fig 11). The pre-processed data effectively reflects the differences in rock properties and segregates five types of facies. However, the facies with the black colour was excluded to avoid the data ambiguities and the other four facies were selected for further analysis. The blue colour facies has been named as Impermeable Reservoir, the green as Potential reservoir, the yellow as Non-Reservoir, and the red as Tight-Reservoir. We can observe that the facies with yellow and green colours constitute the majority of the Lower Goru Formation.

**Fig 12:**
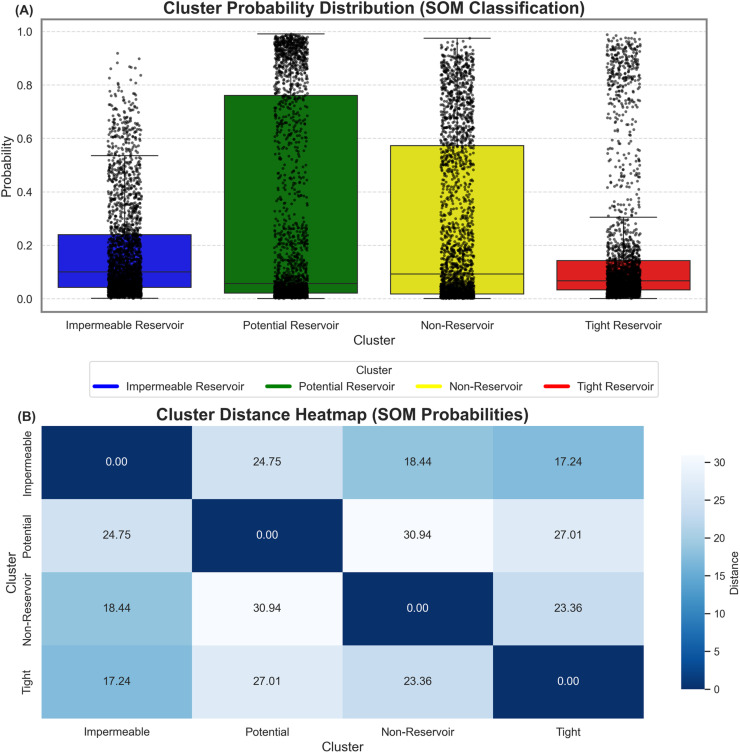
Cluster analysis based on SOM-derived probabilities. **(A**) Box-and-whisker plot depicting the probability distribution of each of the four electrofacies clusters. Higher median probabilities indicate greater cluster confidence and internal coherence, while lower medians suggest less distinct classification boundaries. **(B)** Heatmap of pairwise distances between cluster centroids derived from SOM classification probabilities. Higher values indicate greater dissimilarity between clusters, whereas smaller distances reflect overlapping characteristics. Together, these visualizations validate the distinctiveness and reliability of the identified electrofacies.

Fig 12A depicts the probability distributions for the classified clustered generated through SOM technique. The boxplots, combined with data point distribution plot, highlight the variability and uncertainty captured by SOM. The distribution of probabilities for each facies namely, Impermeable Reservoir, Potential Reservoir, Non-Reservoir, and Tight Reservoir is represented by the boxplots (Fig 12A). The strip plot overlaid on the boxplot provides a granular view depicting the individual data points within each cluster. The boundaries between the clusters indicate the overlap in the classification probabilities, reflecting the inherent heterogeneity and transitional nature of the Lower Goru Formation. The Potential Reservoir has the widest probability range, with the inclusion of other facies. The Tight Reservoir, on the other hand has, the narrowest distribution, indicating its homogeneous properties.

Fig 12B complements this analysis in the form of cluster distance heatmap by quantifying the pairwise distances between cluster centroids based on SOM probabilities. The heatmap depicts that the Potential Reservoir cluster exhibits the greatest distance from the Non-Reservoir cluster, with a value of 30.94, emphasizing that their probabilistic distribution and features are the most dissimilar (Fig 12B). A similar distinction can be observed for the Potential Reservoir and Tight Reservoir, with a value of 27.01. These high values indicate the unique characteristics of the Potential Reservoir cluster setting it apart from the other clusters. On the other hand, the Impermeable Reservoir and Tight Reservoir clusters depict the smallest separation with a value of 17.24, suggesting some shared characteristics or overlapping in their SOM-derived probabilities (Fig 12B). A moderate separation can be observed between the Non-Reservoir and Tight Reservoir clusters, indicated by a value of 23.36, suggesting a partial overlap of features between these clusters (Fig 12B). This overlap of clusters clearly depicts the heterogeneous nature of the Lower Goru Formation, as previously observed.

**Fig 13:**
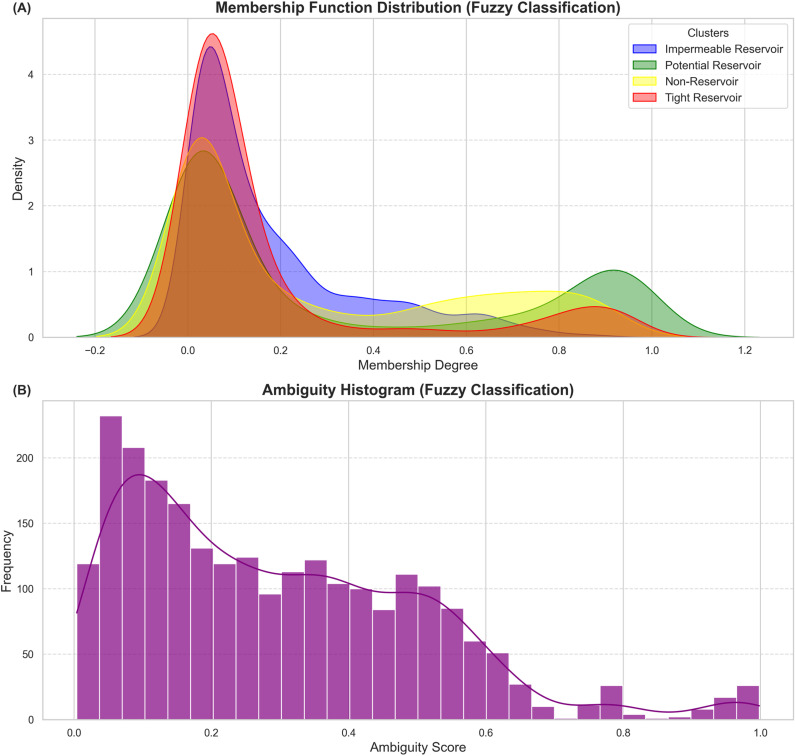
Fuzzy classification analysis. **(A)** Density plot depicting the membership function distribution for the fuzzy classification reflecting the level of association of data points with each cluster. The overlapping areas indicate shared characteristics or transitional zones between clusters. **(B)** Ambiguity histogram for fuzzy classification results depicting ambiguity in the classification of the clusters. The low ambiguity score for most data points suggests a clear cluster assignment.

The probabilistic distribution of cluster membership and classification uncertainty, extracted from the fuzzy classification results is visualized in Fig 13 to emphasize the robust handling of this method. Fig 13A depicts the membership function distribution for the four clusters. The overlapping nature of their density curves illustrates the varying degrees of membership each data point has across clusters. Notably, the distribution peaks of the membership values indicate higher confidence in the classification of each cluster (Fig 13A). The Tight Reservoir shows the highest peak due to its homogeneous nature, while the Potential Reservoir has a dip in the membership peak because of the small intrusions of other facies (Fig 13A). The ambiguity histogram further quantifies the uncertainty associated with the classification, depicting that the most data points are concentrated at lower ambiguity scores (Fig 13A). This implies that most of the points are confidently classified. A gradual decline towards higher ambiguity scores highlights the ability of the fuzzy classification to capture nuanced uncertainties for a subset of points (Fig 13A).

**Fig 14:**
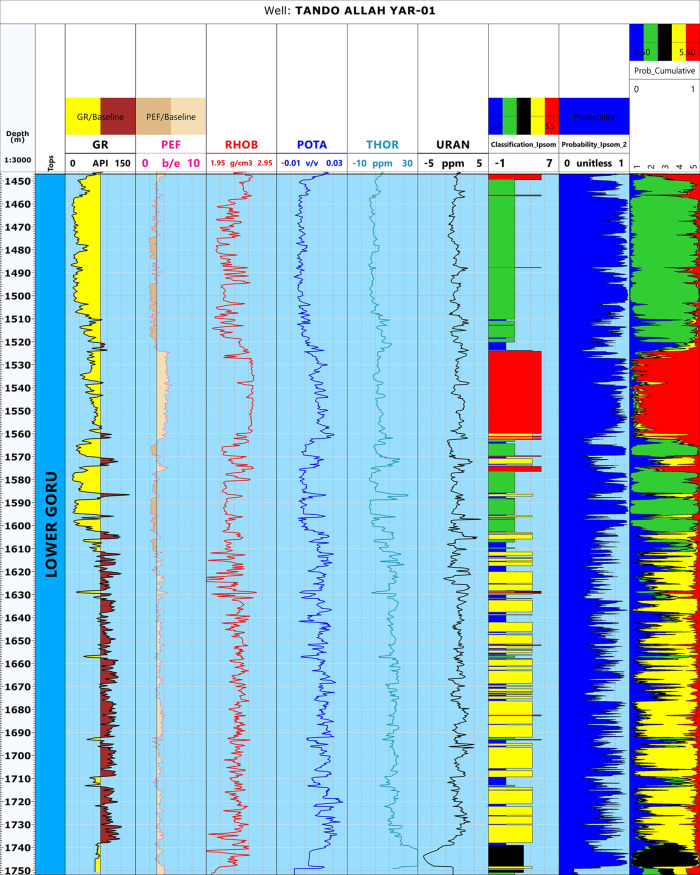
Electrofacies classification for well Tando Allah Yar-01 depicting distinct facies along the depth of Lower Goru Formation.

In the final step of this approach, a log depicting different electrofacies was obtained (Fig 14). This figure shows the electrofacies analysis for the entire depth range of Lower Goru Formation in well Tando Allah Yar-01. The analysis is carried out based on different log responses. Zone 1 indicates the major concentration of reservoir facies, with a minute amount of tight and impermeable reservoir facies. This confirms the results of petrophysical analysis earlier obtained. The tight facies are mainly concentrated in the upper portion of zone 2. In this portion, the density and PEF values are relatively higher. The non-reservoir facies cover the major portion of zone 3 corresponding to high GR values. The Impermeable Reservoir facies is distributed throughout the Lower Goru Formation with an increase in concentration from top to bottom.

## Discussion

The current study aimed to assess the reservoir characteristics and quality of the Lower Goru Formation in the NIM-Tay block of the Lower Indus Basin. This formation is highly heterogeneous in nature, and the quality of the Lower Goru reservoir sands is affected by depositional environments and different diagenetic processes [47,49]. This lithological heterogeneity is primarily driven by variations in sediment supply, depositional energy, and environmental conditions [77]. To study the heterogeneities of the Lower Goru Formation in detail, specifically the role of clay minerals on reservoir quality, an unsupervised machine learning technique was employed to categorize the electrofacies. The electrofacies interpretation was then integrated with petrophysical studies and cross-plot analyses for validation.

### Well logs analysis

The study of different petrophysical parameters of the Lower Goru Formation in the available wells indicates that the presence of shale layers within the sand body negatively impacts reservoir quality (Fig 4). The presence of a thick shale layer in the lower portion of the Lower Goru Formation has significantly reduced reservoir porosity and permeability. However, there is a favorable environment for the accumulation of gas in the upper part of the Lower Goru Formation within the range of 1455–1517 m (Fig 4) (Table 2). This zone is designated as Zone 1 (Fig 4), which is the potential reservoir zone in the study area.

The lithology and type of clay minerals were confirmed through cross-plot analysis. The MN cross-plot analysis indicates that the Lower Goru Formation predominantly consists of quartz and clay minerals, with a minor presence of mica (Fig 5A), thus depicting that the main lithology of the formation is sandstone in the study area. [5], identified sandstone as the primary lithology by using the similar MN cross-plot analysis approach in the Lower Indus Basin. The major data clusters are towards the gas side (Fig 5B), supporting the identification of the potential reservoir zone, as marked on the petrophysical section.

The Th vs.K cross-plot for mineral identification reveals the presence of chlorite, montmorillonite, kaolinite, and mixed clay layer within the entire depth range of Lower Goru Formation (Fig 6A) and chlorite and montmorillonite within the reservoir zone (Fig 6B). A similar approach using the Th vs.K cross-plot technique confirms the presence of chlorite, montmorillonite, kaolinite, and biotite in the Lower Goru Sands in the Lower Indus Basin in the Kadanwari region [5]. The potential reservoir zone, primarily composed of the upper sand body (Zone 1, Fig 4), consists mainly of chlorite (Fig 6B). This indicates that porosity and permeability in this zone are largely preserved by chlorite coatings, as previously reported in the Lower Indus Basin.

### Impact on the reservoir quality

**Fig 15:**
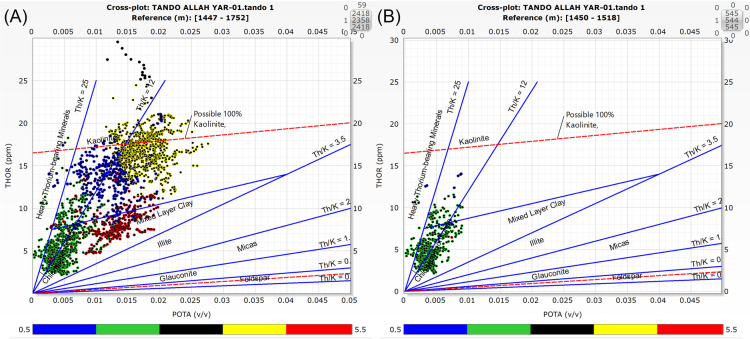
Segregation of clay minerals indicated by Th vs. K cross-plot for well Tando Allah Yar-01 highlighted in accordance with electrofacies classification. (**A**) depicts clay mineral facies for the entire depth range of Lower Goru Formation. (**B**) depicts the clay minerals facies for reservoir zone.

**Fig 16:**
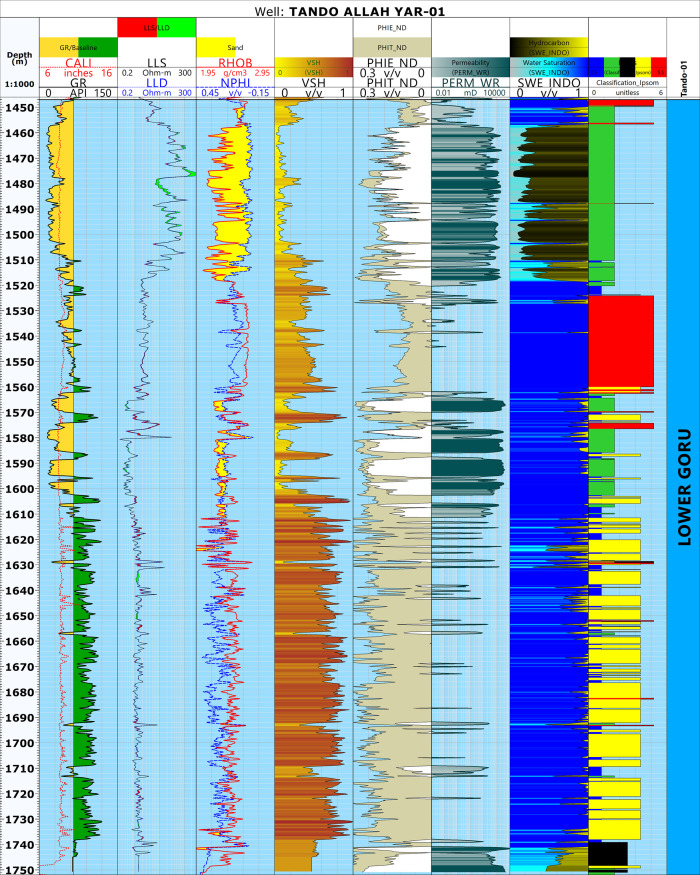
Electrofacies classification along with petrophysical analysis of Tando Allah Yar -01 for reservoir quality prediction.

Finding the most productive lithofacies has several benefits, such as predicting reservoir quality. Reservoir characteristics such as porosity and permeability vary among different lithofacies and rock types [54]. The identification of lithofacies facilitates the assessment of reservoir quality and potential hydrocarbon productivity [24]. The correlation of facies with minerals and petrophysical properties such as porosity and permeability allows a more accurate analysis of the reservoir quality [99]. Therefore, to assess how the identified facies influence reservoir quality, we correlated them with the corresponding minerals and the petrophysical results (Figs 15 and 16).

In Fig 15, the electrofacies are superimposed onto the Th vs. K cross-plot to identify the different types of clay minerals within each electrofacies. This figure clearly indicates that each electrofacies comprises a combination of different clay minerals. The cross-plot analysis coupled with electrofacies classification indicates the presence of four types of clay minerals, i.e., chlorite, montmorillonite, kaolinite, and mixed clay layer within the Lower Goru Formation (Fig 15A and 15B) within the identified facies (Fig 14). The Th vs.K cross plot methodology, combined with electrofacies classification, provides a quantitative tool for mineralogical differentiation rather than depending entirely on qualitative interpretations.

Fig 16 incorporates the petrophysical analysis results with the electrofacies classification to provide a more comprehensive understanding of each facies by analyzing porosity, permeability, and water saturation within each zone. The Impermeable Reservoir Facies indicates high concentration of kaolinite with very minor concentration of chlorite and montmorillonite (Fig 15A), a composition that significantly reduces the reservoir quality in this zone. The effective porosity and permeability values corresponding to this facies are 8% and 108.36 mD, respectively. The Non-Reservoir Facies also has high concentration of kaolinite (Fig 15A) with an effective porosity of 5% and permeability of 6.65mD. The corresponding porosity and permeability values within these two facies are low, indicating the negative impact of kaolinite on the reservoir quality (Fig 16, Table 3). However, a notable observation from this study is that the Impermeable Reservoir Facies has slightly higher average permeability and porosity (8% and 108.36 mD, respectively) than the Non-Reservoir Facies (5% and 6.65 mD), implying that even a minor presence of chlorite can mitigate the negative effects of kaolinite (Table 3) [100]. reported authigenic kaolinite in basal sands of Lower Goru Formation as pore-filling clay that lowers the interparticle porosity. This is an important result because prior research [100] linked kaolinite to substantial porosity loss in the Lower Goru Formation without considering the potential buffering impact of chlorite. The porosity and permeability reduction in these facies might be due to the cementation of kaolinite by filling and lining the pore spaces and pore throats as this mineral is reported as porosity and permeability reduction mineral in sandstone reservoirs by numerous researchers worldwide [6,7,14–16,101].

**Table 3:** Average petrophysical values for each electrofacies in Well Tando Allah Yar-01.

Electrofacies	PERM_WR (mD)	PHIE_ND (%)	PHIT_ND (%)	SWE_INDO (%)	VSH (%)
			%	%	%
**Impermeable Reservoir**	108.36	8	20	96	52
**Potential Reservoir**	888.87	15	19	54	17
**Non-Reservoir**	6.65	5	21	100	70
**Tight Reservoir**	28.25	4	12	98	38

The reservoir in our case is a major sand body classified as the Potential Reservoir Facies (upper sands, Zone 1) (Fig 4), which indicates the presence of chlorite and montmorillonite with a low concentration of kaolinite (Fig 15B). Fig 16 clearly indicates that the porosity (15%) and permeability (888.87 mD) are higher (Table 3) within the Potential Reservoir Facies, thus indicating that the chlorite and montmorillonite are preserving the porosity and permeability. The presence of chlorite in Lower Goru Sandstone acts as a porosity and permeability preserving mineral which is supported by earlier studies [51,52] in the basin. The fundamental porosity is retained in the early stages of authigenic chlorite coating in the Lower Goru Formation [49]. According to [51], quartz cementation was prevented by well-developed chlorite rims, which also retained porosities up to 20% and are responsible for good permeabilities in the Lower Goru Formation. The previous studies in the Lower Indus Basin were based on conventional petrographic and diagenetic approaches and our results from machine learning further validates and refines those findings.

The Potential Reservoir Facies (upper sands, Zone 1) contains a small amount of montmorillonite in the reservoir zone (Fig 15B), which typically reduces the porosity and permeability of the reservoir. Montmorillonite is a member of the smectite family, and it grows as small crystals that fill the pores between grains [102] and generally shows a swelling property when exposed to water [12,103]. In Lower Indus Basin, the smectite clays in the form of mix layer of Illite-smectite are responsible for lowering the quality of the Lower Goru reservoir [49]. However, in this case, the high porosity (15%) and permeability (888.87 mD) values were observed in the presence of montmorillonite. The studies of [6,7] also shows a positive effect of montmorillonite on the permeability by coating the pores in clastic reservoirs deposited in the same shallow marine environment as that of Lower Goru Formation. Since the Lower Goru Formation was deposited in a marine to shallow marine environment [47,49,104], can therefore have the same implications for the presence of montmorillonite. Therefore, the high porosity and permeability values in our reservoir despite the presence of montmorillonite might be due to the coatings.

The Tight Reservoir Facies show the presence of mixed layer clay (Fig 15A) corresponding to reduced porosity (4%) and permeability (28.25 mD) values (Fig 16, Table 3). Mixed-layer clay is formed due to interlayering of different clay minerals within a single structure [105]. These layers are mainly composed of illite-smectite and chlorite-smectite [13]. The mixed layers indicate the presence of illite and illite-smectite, which constrict the pore throat and have a negative impact on the reservoir properties [49]. The diverse composition of clay minerals within the identified electrofacies offers a novel perspective as previous research works are mainly focused on the separate response of each clay mineral. This is helpful in analyzing the effect of the highly heterogeneous behavior of clay minerals on the reservoir quality of sandstone formations. A minor concentration of porosity preserving or non-preserving minerals can significantly alter the reservoir quality.

For a better contextualization of these findings, it is useful to compare this workflow with previous studies that have used machine learning-based methods for electrofacies and lithofacies classification in the Lower Indus Basin. Several recent studies have applied machine learning techniques for lithofacies and electrofacies classification in the Lower Indus Basin, highlighting their utility in delineating reservoir characteristics. For example [25], utilized SOM based clustering to segregate reservoir facies from non-reservoir facies using petrophysical parameters in Zamzama Gas Field. [53] and [54] identified depositional facies and reservoir rock types using machine learning models, namely K-means and Density-Based Spatial Clustering of Applications with Noise, in the Lower Goru Formation. These works demonstrate the efficacy of unsupervised learning such as SOM, supported by clustering and petrophysical validation, yield reliable classifications, particularly for delineating pay zones. Similarly [56], and [106] applied various machine learning algorithms to predict lithofacies in tight sandstone reservoirs, focusing on improving model interpretability and spatial continuity through geostatistical simulations and Shapley Additive Explanations analysis.

Unlike previous studies which primarily focused on pay zone predictions or facies classification based on rock types, the present study introduces a workflow that uniquely integrates PCA-based preprocessing with SOM and fuzzy classification to delineate clay mineral-based electrofacies. This approach enables a more detailed understanding of the impact of mineralogical constituents on reservoir quality. While [48] derived electrofacies solely from GR logs to study depositional environments, our approach is explicitly focused on the influence of clay minerals such as chlorite, kaolinite, and montmorillonite on the reservoir. Moreover, the workflow includes a validation framework that quantitatively links electrofacies to porosity, permeability, and mineralogical cross-plots, offering a comprehensive and reproducible framework for reservoir quality assessment in Lower Indus Basin and other similar depositional settings.

**Fig 17:**
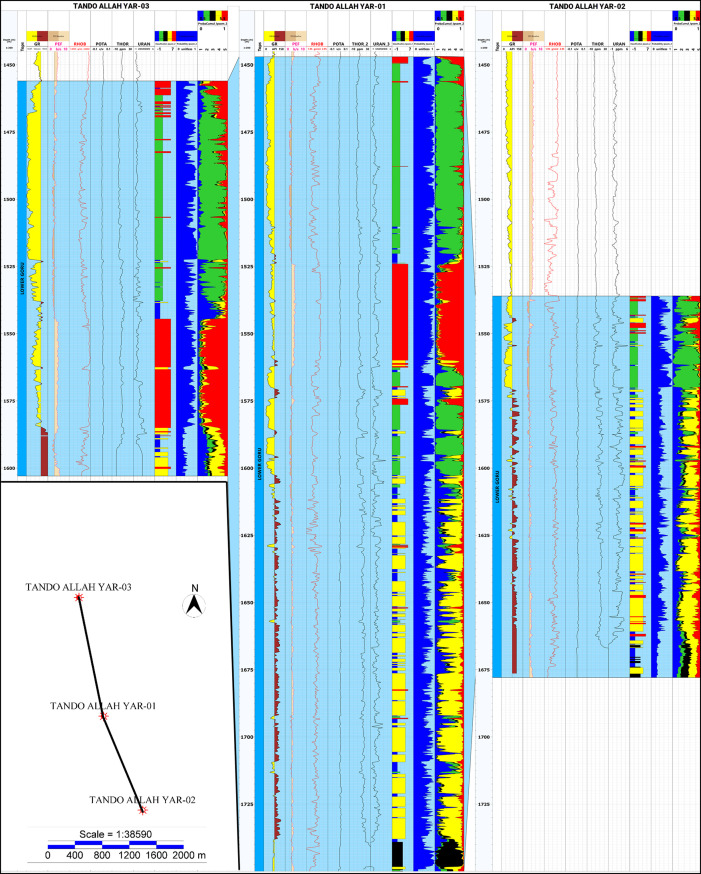
Electrofacies classification of all three wells, i.e., Tando Allah Yar-01, Tando Allah Yar-02, and Tando Allah Yar-03.

The opted machine learning methodology provides a solid foundation for precisely analyzing the influence of clay minerals on reservoir quality by utilizing broader datasets with ease. The model was trained for well Tando Allah Yar-01 and then applied on the other two wells, offering a more regional understanding of reservoir heterogeneity and stratigraphic continuity. Fig 17 shows the combined electrofacies classification for wells Tando Allah Yar-01, Tando Allah Yar-02, and Tando Allah Yar-03, depicting a stratigraphic coherence, indicated by the similar distribution of facies along the well. The continuity of Facies 2 can be observed in the wells Tando Allah Yar-03 and Tando Allah Yar-01; however, the reservoir quality is poor Tando Allah Yar-02, which might be due to the presence of faults as the area is under the influence of an extensional regime (Fig 17). Furthermore, the high concentration of Non-Reservoir Facies in the lower portion of the wells Tando Allah Yar -3 and 02 suggests an increase in clay content (kaolinite) as we move from north to south (Fig 17).

### Practical implications and methodological advancement

The integration of unsupervised machine learning techniques with petrophysical and mineralogical analysis offers a significant advantage over conventional reservoir quality assessment methods. Traditional approaches often rely on qualitative interpretations from core samples or well log studies, which can prove to be time-consuming, expensive, and spatially limited. In contrast, the electrofacies classification approach used in this study provides a rapid, reproducible, and precise lithofacies discrimination across the entire formation. The method allows a better understanding of clay mineral variability and its direct impact on porosity and permeability. Using conventional techniques alone, assessing subtle implications of mineral variation, as in the case of the marginal improvements in reservoir quality due to the presence of minor chlorite content, seems difficult to detect.

In terms of reservoir exploitation, the adopted methodology supports efficient decision-making in field development planning. Identifying chlorite-dominated zones, for example, as porosity- preserving targets can help optimize perforation intervals and reduce costs associated with unnecessary stimulation in poor-quality zones. Furthermore, the model’s successful application across multiple wells (Fig 17) demonstrates its robustness and potential usage in other stratigraphic units with similar depositional environments, such as other marine-influenced sandstone reservoirs within the Lower Indus Basin or similar settings globally. This not only contributes to better reservoir heterogeneity assessment but also aligns with the field’s ongoing efforts to integrate artificial intelligence and data-driven workflows for improved reservoir characterization.

This study demonstrates that machine learning-based electrofacies classification offers a significant improvement in interpretability and consistency over conventional petrophysical techniques for capturing subtle mineralogical variations that are prone to be overlooked in traditional analyses. By integrating dimensionality reduction with unsupervised clustering, the approach enhances classification accuracy while reducing subjectivity, making it a robust alternative for reservoir quality assessment. However, the approach may require careful tuning of clustering parameters and relies upon the input log data quality, which could limit its efficacious usage with poor quality data.

## Conclusion

The obtained results demonstrate the presence of distinct types of clay minerals with a varying impact on the reservoir quality of the Lower Goru Formation.

1. The analysis of SOM and fuzzy classification results confirms the heterogeneous nature of the Lower Goru Formation highlighting the impact of clay minerals on the reservoir quality of the formation.
2. Based on the petrophysical analysis, Zone 1 was marked as the possible hydrocarbon producing zone in the Lower Goru Formation (depth 1455–1517 m). The effective porosity and permeability values of this portion are 13.9% and 434.7 mD respectively, while water saturation is 27.3%, indicating a satisfactory reservoir quality and hydrocarbon potential.
3. Electrofacies classification coupled with the petrophysical analysis suggests the presence of four distinct facies within the Lower Goru Formation, namely Impermeable Reservoir (blue), Potential Reservoir (green), Non-Reservoir (yellow), and Tight Reservoir (red).
4. Petrophysical studies coupled with cross-plot analysis and electrofacies classification identified four types of clay minerals within the Lower Goru Formation namely chlorite, montmorillonite, kaolinite, and mixed-layer clay.
5. The Potential Reservoir Facies mainly consists of chlorite and montmorillonite, while the Impermeable Reservoir shows a high concentration of kaolinite with minor amount of chlorite and montmorillonite. The Non-Reservoir Facies comprise primarily of kaolinite, while mixed layer clays are observed in the Tight Reservoir Facies.
6. The combined analysis suggests that chlorite and montmorillonite in the potential reservoir facies are the major porosity and permeability preserving minerals with average effective porosity of 15% and permeability as 888.87 mD.
7. Kaolinite (average effective porosity 5% and permeability 6.65 mD) and mixed layer clay (average effective porosity 4% and permeability 28.25 mD) reduce the reservoir quality by lowering the porosity and permeability values. The presence of these two minerals is the primary cause of reduced reservoir quality in the lower portion of the Lower Goru Formation in this area.

## Supporting information

S1 File S1_ Tando Allah Yar 1,2,3. (RAR)
